# The role of geriatric oncology in the care of older people with cancer: some evidence from Brazil and the world

**DOI:** 10.1590/1806-9282.2024S118

**Published:** 2024-06-07

**Authors:** Theodora Karnakis, Polianna Mara Rodrigues de Souza, Ana Lumi Kanaji, Lessandra Chinaglia, Mirella Rebello Bezerra, Olga Laura Sena Almeida

**Affiliations:** 1 Universidade de São Paulo, Cancer Institute of the State of São Paulo – São Paulo (SP), Brazil.; 2 Teaching and Research Institute, Sírio-Libanês Hospital – São Paulo (SP), Brazil.; 3 Brazilian Society of Geriatrics and Gerontology, Geriatric Oncology Commission – Brazil.; 4 São Paulo Medical School, Laboratory of Medical Investigation in Aging (LIM 66) – São Paulo (SP), Brazil.; 5 Albert Einstein Israelite Hospital – São Paulo (SP), Brazil.; 6 Oncology Center of Prevent Senior – São Paulo (SP), Brazil.; 7 Institute of Integral Medicine Professor Fernando Figueira – Recife (PE), Brazil.

## EPIDEMIOLOGY OF CANCER AND AGING

According to the World Health Organization (WHO), cancer is defined as a large group of malignant diseases that have the common characteristic of disordered and abnormal cell growth, which can invade adjacent structures or spread through metastases. According to WHO data, in 2018, 17 million new cases and 9.6 million deaths from cancer were recorded worldwide, generating a great impact not only physically but also emotionally and financially on individuals, families, communities, and health systems^1^. In Brazil, 704,000 new cases of cancer are expected for each year of the 2023–2025 triennium, with emphasis on the South and Southeast regions, which account for around 70% of cases. Among the most common malignant neoplasms in Brazil, non-melanoma skin neoplasms are the most common (31.3% of total cases), followed by breast neoplasms (10.5%), prostate (10.2%), colon and rectum (6.5%), lung (4.6%), and stomach (3.1%)^1,2^.

Cancer is considered a public health problem because it is the second leading cause of mortality in the world and, consequently, one of the main barriers to increasing the population's life expectancy. Furthermore, the impact of its incidence and mortality is increasing rapidly on the global stage due to the demographic and epidemiological transition that the world is going through, with the increase in population aging, especially in developing countries such as Brazil^2,3^.

Age is known to be an important risk factor for the development of cancer. It is known that 56% of cancer diagnoses and 70% of all deaths from oncological diseases occur in older people^4^. Furthermore, cancer is the second leading cause of death in women and men aged 60–79 years. The average age of cancer diagnosis is 68 years, and the incidence of cancer increases with age, with an 11 times greater risk of developing cancer in people over 65 years of age. It is estimated that, by 2040, the incidence of cancer will double in the older population over 65 years of age, with an even greater increase in incidence among octogenarians^5^.

Faced with this increase in the incidence of cancer in the older, geriatric oncology has become a field in full expansion, nationally and internationally. Guidelines for acting in geriatric oncology have been recommended by the main international oncology societies, bringing the importance of discussing the topic between geriatrics and oncology societies in Brazil and health professionals who assist the older population with cancer^6,7^.

## HISTORY OF GERIATRIC ONCOLOGY AND ITS CHALLENGES

The history of geriatric oncology is evident in the first conference focused on Cancer in the older, in 1983, organized by Rosemary Yancik (PhD) and Paul Carbone (MD) at the National Institutes of Health^4,8^. Subsequently, in 1990, a group of seven medical oncologists from Guy's Hospital in London published the paper "Cancer in the older: Why So Badly Treated?" in The Lancet journal^9^. The article detailed the enormity of the problem and inspired many young investigators and clinicians to join the field of geriatric oncology. In 2000, the International Society of Geriatric Oncology (SIOG) was founded.

International Society of Geriatric Oncology is a multidisciplinary membership-based society with members engaged in more than 80 countries around the world. Their network includes geriatricians, medical oncologists, surgical oncologists, radiation oncologists, anesthesiologists, nurses, and allied health professionals. Ever since, SIOG has established a long-standing history of implementing programmatic activities in the field of geriatric oncology in four strategic directions: education, clinical practice, research, and collaborations and partnerships^10^. Since 2010, the Journal of Geriatric Oncology is the official journal of SIOG. It is an international multidisciplinary journal that is focused on advancing research in the pathogenesis, biology, treatment, and survivorship issues of older adults with cancer. The journal covers all aspects of geriatric oncology, from basic scientific research through to clinical research, as well as research that is relevant to education and policy development, and it has a high impact factor of 3.9^10^.

The literature raises many challenges in covering the care of older people with cancer. Globally, the geriatric training of healthcare personnel is regulated by diverse organizations, leading to high variability in curriculum and certificates^11^. In order to solve this, efforts to put together a homogeneous core curriculum in geriatric medicine and to include geriatric oncology in training programs have been undertaken by several medical societies around the world. The American Society of Clinical Oncology (ASCO) and the European Society of Medical Oncology (ESMO) included recommendations for core geriatric oncology training as a part of the global curriculum in medical oncology^11,12^. Likewise, the European Oncology Nursing Society has also published recommendations for the creation of a homogeneous curriculum for cancer in older people^13^.

In Brazil, many initiatives in geriatric oncology began to appear in the last two decades. Most of them have been undertaken in the southeast of the country and in the last 5 years expanding to the center and northeast of the country. In 2012, the Cancer Institute of the State of São Paulo (ICESP) established a geriatric oncology program that employs four geriatricians full-time and provides training for medical residents and fellows in both geriatrics and oncology^7,14^. In 2013, the geriatric oncology outpatient service was also created at the Clinics Hospital of the Faculty of Medicine of Ribeirão Preto to care for older people with cancer and support medical oncologists in therapeutic decision-making. Both services are linked to the University of São Paulo and the public health system and develop assistance, teaching, and research activities. There are other geriatric oncology clinics that are located at private hospitals in São Paulo, such as Sírio-Libanês Hospital, Israelita Albert Einstein Hospital, A.C. Camargo Cancer Center, and Prevent Senior Health System, or in public hospitals as Institute of Integral Medicine Professor Fernando Figueira (IMIP) that is located in Recife, in the northeast of the country. Most of these clinics follow an interconsultation model, in which specialized geriatricians perform geriatric assessment-based recommendations to referring oncologists^14^. Recently, a national survey aimed at understanding the geriatric knowledge of oncology professionals in the country was designed and administered using a web-based platform. Notably, 60% of respondents reported having a population of older patients in their clinics between 26 and 50%, and 65% of them believed that chronological age should not be the single factor determining treatment initiation in an older patient. However, most participants (70%) didn't have a geriatrics program at their institution^15^.

In 2011, with the support of the Brazilian Society of Clinical Oncology (SBOC) and the Brazilian Society of Geriatrics and Gerontology (SBGG), the first international symposium of geriatric oncology took place in São Paulo. In 2012, the first national book on geriatric oncology was published. However, just in 2020, a commission of geriatricians who work caring the older people with cancer across the country was formed by SBGG with the main objective of developing a competency matrix that determines the skills required for geriatricians regarding the care of older with cancer, especially geriatricians in training, to be used in various geriatrician training centers across the country. The objective of this matrix would be to guarantee the quality of care and safety of older patients with cancer. The secondary objectives of this commission were disseminating basic scientific knowledge in geriatric oncology to health professionals who treat the older population, promoting educational actions on prevention, diagnosis, and treatment of oncological diseases in this population, and promoting the integration between the different societies involved in the care of older people with cancer in the country.

The implementation of a geriatric oncology service is challenging in both high-income countries and low-income countries, as there is a significant demand for economic and human resources needed for structure and training^14^. A recent publication by the geriatric oncology service of the ICESP raises the importance of initiatives for better interdisciplinary integration between the specialties of geriatrics, oncology, radiology, and surgery in the treatment of older people with cancer^16^.

## THE ROLE OF THE GERIATRICIAN IN THE CARE OF THE OLDER WITH CANCER

Management of older patients with cancer is often complex. As the aging process is multifactorial and does not occur in the same way in all individuals, the older population is quite heterogeneous in several aspects. Thus, chronological age does not reflect biological or functional age and should not be the only factor to be considered when making decisions about cancer treatment for older people^6^.

The impact of cancer and its treatment on the older can be significant, depending on factors such as functionality, cognition, emotional profile, socioeconomic status, nutritional status, presence of comorbidities, and drug use profile, in addition to individual values and preferences^17^.

Some studies have shown that traditional functional assessments in oncology, such as Karnofsky performance status (KPS) or Eastern Cooperative Oncology Group (ECOG) performance status scores, are not accurate enough to predict outcomes in older adults with cancer^18,19^. Thus, the Comprehensive Geriatric Assessment (CGA), a systematized process well known to geriatrics and gerontology professionals for the multidimensional assessment of the older, has come to be widely studied in the scenario of cancer treatment and in aid to decision-making in geriatric oncology, with detailed evaluation of domains such as functionality, cognition, comorbidities, medications in use, nutritional status, psychological status and social support, as well as estimates of life expectancy and risk of toxicity to chemotherapy^17,20,21^. The main instruments used in the implementation of the AGA are listed in Table 1.

**Table 1:** Domains in a comprehensive geriatric assessment.

Domain	Geriatric assessment tool	Intervention for positive finding
Functional status	Self-reported: – Activities of daily living (ADLs) – Instrumental activities of daily living (IADLs) – Falls Objective tests: – Timed up and go test (TUG) – Gait speed – Short physical performance battery (SPPB)	– Mobility and health aids – Home safety equipment – Promote physical activity – Physical therapy and rehab
Comorbidity	– Charlson Comorbidity Index (CCI) – Cumulative index rating scale (CIRS) – Adult comorbidity evaluation-27 (ACE-27)	– Comorbidity management – Referral to geriatrician – Clarify goals of care
Social functioning and support	– Medical outcomes study (MOS) survey – RAND health survey	– Consult social work – Consult financial services
Cognition	– Mini-cog – Mini-mental state examination (MMSE) – Blessed orientation memory concentration (BOMC) test – Montreal Cognitive Assessment (MoCA)	– Counseling – Assess inappropriate medications – Evaluate capacity – Referral to geriatric neuropsychologist
Psychological	– Mental health inventory distress thermometer – Geriatric Depression Scale-4 (GDS-4) – Patient health questionnaire-2 (PHQ-2)	– Cognitive behavioral therapy – Non-pharmacologic approaches (meditation) – Antidepressants – Referral to a geriatric psychiatrist
Nutrition	– Body mass index (BMI) – Mini nutritional assessment (MNA) – Malnutrition universal screening tool (MUST)	– Address factors contributing to malnutrition – Address chemotherapy-induced adverse effects such as nausea/vomiting – Oral care – Supplemental nutrition – Refer to a dietician
Polypharmacy	– Beers criteria – Medication – Appropriateness Index (MAI) – STOPP/START criteria	– Medication reconciliation – Evaluate adherence – Evaluate drug interactions – Deprescribing – Home health for medication management

Adapted from Kapoor and Arora^20^.

The use of CGA in oncology has been shown to be beneficial in several studies, allowing better identification of areas of greater vulnerability or fragility and helping to predict the risk of negative outcomes, toxicity to cancer treatment, functional impairment, and mortality^18,20^.

Functional dependence on instrumental activities of daily living is associated with worse outcomes throughout treatment and reduced overall survival in several types of cancers. Among the negative outcomes found are a higher risk of additional functional loss and toxicity to treatment, as well as a higher rate of treatment interruption. Reduction in gait speed is associated with a higher risk of mortality, unplanned hospitalizations, and visits to urgent and emergency departments. Decreased handgrip strength is associated with worse survival^18,20,22^. In an important study that aimed to evaluate the main risk factors for chemotherapy toxicity, the measures of functional capacity were predictors of high risk for falls in the last 6 months, limitations to walk one block, need for assistance to take medications, and decrease in social activities^19^.

Cognitive decline in older adults is associated with a higher risk of all-cause mortality, including cancer mortality, and is associated with poor medication adherence in any health setting. Low scores on cognitive assessment tests, such as the Mini-Mental State Examination (MMSE), have been shown to be an independent risk factor for unplanned hospitalization and discontinuation of cancer treatment in several types of cancer^18,20,22^.

Malnutrition and increased risk of nutritional impairment identified by the Mini Nutritional Assessment (MNA) scale, especially reduced food intake in the last 3 months, are associated with a higher risk of toxicity and low tolerance to chemotherapy, early discontinuation of cancer treatment, loss of functionality, prolonged hospitalizations, impaired quality of life, and lower survival^18,20,22^.

In older cancer patients, comorbidities can complicate the diagnosis and treatment of cancer. The presence of comorbidities is associated with worst survival in older cancer patients, a higher risk of toxicity to chemotherapy, a higher rate of hospitalization, and early discontinuation of cancer treatment^18,20,23^.

Depression is quite prevalent in older people with cancer and can affect up to 30% of patients. It may be associated with a higher risk of functional and cognitive impairment during cancer treatment^18,20,22,23^.

The presence of polypharmacy in older adults with cancer is associated with a higher risk of falls, frailty, postoperative complications, chemotherapy toxicity, increased healthcare costs, and mortality. The absence of social support or insufficient support was identified as a predictor of mortality in older adults with cancer^18,20,22^.

Recent randomized controlled trials evaluating the impact of CGA-guided geriatric interventions have demonstrated important benefits such as reduced severe toxicity to chemotherapy, reduced rates of treatment interruption due to toxicity, reduced unplanned hospitalization, and higher rates of advance directives. Thus, the results of the AGA help not only to inform patients and families about the risks and benefits of cancer treatment, aiding in shared decision-making processes, but also to promote appropriate interventions, counseling, and referrals, improving the journey of older patients throughout their cancer treatment^21,23,24^.

## GERIATRIC ONCOLOGY PERSPECTIVES

Despite the evidence about the benefits of using the CGA in the care of patients with cancer, there are points to explore and challenges to overcome. Most studies include patients with solid tumors and lymphoma who received cytotoxic treatment and there is evidence about the role of the CGA in the context of immunotherapy, targeted therapy, bone marrow transplant, and cell therapy. Furthermore, the CGA is important in the beginning of the cancer treatment. However, no data indicate what is the best interval time to reassess the patient during the cancer treatment^21^.

In addition to the complexity of older patients and the peculiarities of cancer treatment in this population, oncologists and geriatricians still need to deal with the challenge of little scientific evidence related to the treatment of older people with cancer, especially those over 75 years of age. There is still a significant underrepresentation of older people in clinical trials that establish standards for oncological care, making it difficult to extrapolate results to the older population^25^. In the coming years, it is expected that more older people will be included in clinical trials and that functional and quality of life outcomes will also be evaluated, in addition to survival^26^.

From a practical point of view, there are barriers to implementing geriatric oncology services such as the lack of qualified geriatricians and the lack of oncologist's knowledge about the role of geriatricians in the care of older patients with cancer. According to the National Council of Medicine, as of 2014, there were 1405 geriatricians practicing throughout Brazil, which translates to an average of 0.7 geriatricians per 100,000 inhabitants. At the same time, the number of certified oncologists was 3409, translating into an average of 1.7 oncologists per 100,000 inhabitants^27^.

Because there are multiple tools, geriatric scales, and recommendations, it is mandatory to develop a standard objective language to avoid ambiguous interpretations that may hinder information integration and patient care^28^.

## CONCLUSION

As life expectancy increases, the number of older patients with cancer will certainly continue to rise in Brazil and healthcare systems throughout the country will be forced to respond to this situation in a timely manner. Although, in Brazil, geriatric oncology is still at a very early stage, there is a great opportunity to develop resources and research for the creation and implementation of novel models of care that meet the needs of the older population with cancer in the country.

Given the above, geriatric oncology emerged as an area of activity for oncologists and geriatricians to provide better care for older people with cancer and has been growing exponentially in recent years. This is an area of activity that aims to develop more integrated therapeutic strategies that allow the creation of an individualized geriatric care plan. This integration allows for a more careful and comprehensive assessment of older people with cancer and ensures that age is not a factor of discrimination in access to oncological treatment, in addition to improving clinical outcomes and quality of life through geriatric interventions that, many sometimes, they would not be performed or would go unnoticed in the usual oncological evaluation.
